# Supplementary material for: Prognostic Factors of Renal Outcomes after Heart Transplantation: A Nationwide Retrospective Study
Source: J Clin Med. 2021 Oct 30;10(21):5110. doi: 10.3390/jcm10215110 (PMC8584983; doi:10.3390/jcm10215110)
Supplement: Supplementary file 1 [file jcm-10-05110-s001.zip › jcm-1417967-supplementary.pdf]

Table S1. List of medications ATC codes.

Name	ATC code
**Vasopressor drugs**	
Dopamine	C01CA04
Norepinephrine	C01CA03
Epinephrine	C01CA24
Lidocaine, combinations	N01BB52
Vasopressin	H01BA01
Dobutamine	C01CA07
Milrinone	C01CE02
**Angiotensin II receptor blocker**	
Losartan	C09CA01
Eprosartan	C09CA02
Valsartan	C09CA03
Irbesartan	C09CA04
Candesartan	C09CA06
Telmisartan	C09CA07
Olmesartan medoxomil	C09CA08
Fimasartan	C09CA10
Angiotensin II antagonists and diuretics	C09DA
Losartan and diuretics	C09DA01
Eprosartan and diuretics	C09DA02
Valsartan and diuretics	C09DA03
Irbesartan and diuretics	C09DA04
Candesartan and diuretics	C09DA06
Telmisartan and diuretics	C09DA07
Olmesartan medoxomil and diuretics	C09DA08
Valsartan and amlodipine	C09DB01
Olmesartan medoxomil and amlodipine	C09DB02
Telmisartan and amlodipine	C09DB04
Losartan and amlodipine	C09DB06
Valsartan and lercanidipine	C09DB08
Olmesartan medoxomil, amlodipine and hydrochlorothiazide	C09DX03
Valsartan and sacubitril	C09DX04
HMG CoA reductase inhibitors, other combinations	C10BX
Rosuvastatin and valsartan	C10BX10
**Angiotensin-converting enzyme inhibitor**	
ACE inhibitors, plain	C09AA
Captopril	C09AA01
Enalapril	C09AA02
Lisinopril	C09AA03
Perindopril	C09AA04
Ramipril	C09AA05
Quinapril	C09AA06
Benazepril	C09AA07
Cilazapril	C09AA08
Moexipril	C09AA13
Temocapril	C09AA14
Zofenopril	C09AA15
Imidapril	C09AA16
Captopril and diuretics	C09BA01
Enalapril and diuretics	C09BA02
Lisinopril and diuretics	C09BA03
Perindopril and diuretics	C09BA04
Ramipril and diuretics	C09BA05
Ramipril and felodipine	C09BB05

Table S2. Characteristics of heart transplantation patients with and without chronic kidney disease after heart transplantation.

	Without CKD (N = 457)	With CKD (N = 104)	*P* value
**Age (year), median (IQR)**	45 (37–58)	49 (53–61)	0.009
**Sex, male**	299 (65.4)	79 (76.0)	0.039
**Charlson's index, median (IQR)**	3 (2–4)	3 (2–5)	0.388
**Comorbidity**			
Peripheral vascular disease	66 (14.4)	10 (9.6)	0.194
Cerebrovascular disease	65 (14.2)	15 (14.4)	0.958
Chronic pulmonary disease	209 (45.7)	48 (46.2)	0.938
Connective tissue disease	21 (4.6)	4 (3.8)	0.738
Liver disease	163 (35.7)	40 (38.5)	0.593
Diabetes	25 (5.5)	12 (11.5)	0.194
Hypertension	381 (83.4)	82 (78.8)	0.273
**History of transplantation**	9 (2.0)	1 (1.0)	0.697
**Mechanical ventilation**	92 (20.1)	35 (33.7)	0.003
**ECMO**	89 (19.5)	30 (28.8)	0.035
**Renal replacement therapy**			0.018
No	383 (83.8)	76 (73.1)	
1–21 days	60 (13.1)	20 (19.2)	
>21 days	14 (3.1)	8 (7.7)	
**Inotropic or vasopressors**	196 (42.9)	32 (30.8)	0.023
**ACEi/ARB**	156 (34.1)	29 (27.9)	0.221
**ICU length of stay, (median, IQR)**	7 (5–15)	10 (6-20)	<0.001
**Hospital length of stay, (median, IQR)**	60 (36–83)	48 (33–73)	0.039

Abbreviations: ECMO; extracorporeal membrane oxygenation, ACEi; angiotensin-converting enzyme inhibitor, ARB; angiotensin II receptor blocker, ICU; intensive care units; Values are the median (interquartile range) or n (%).

Table S3. Characteristics of heart transplantation patients according to hospital mortality.

	Overall (N = 736)	Survival (N = 679)	Death (N = 57)	*P*-value
**Age (year), median (IQR)**	52 (40–60)	52 (40–60)	54 (47–60)	0.15
**Sex, male**	501 (68.1)	462 (68.0)	39 (68.4)	0.95
**Charlson's index, median (IQR)**	3 (2-5)	3 (2-5)	4 (2-5)	0.55
**Comorbidity**				
Peripheral vascular disease	112 (15.2)	101 (14.9)	11 (19.3)	0.37
Cerebrovascular disease	114 (15.5)	105 (15.5)	9 (15.8)	0.95
Chronic pulmonary disease	350 (47.6)	323 (47.6)	27 (47.4)	0.98
Connective tissue disease	36 (4.9)	34 (5.0)	2 (3.5)	0.99
Liver disease	280 (38.0)	259 (38.1)	21 (36.8)	0.85
Diabetes	79 (10.7)	71 (10.5)	8 (14.0)	0.40
Renal disease	118 (16.0)	102 (15.0)	16 (28.1)	0.01
Hypertension	615 (83.6)	569 (83.8)	46 (80.7)	0.54
**History of heart transplantation**	13 (1.8)	12 (1.8)	1 (1.8)	0.99
**Mechanical ventilation**	216 (29.3)	167 (24.6)	49 (86.0)	<0.001
**ECMO**	182 (24.7)	147 (21.6)	35 (61.4)	<0.001
**Renal replacement therapy**				<0.001
No	531 (72.1)	526 (77.5)	5 (8.8)	
1–21 days	143 (19.4)	115 (16.9)	28 (49.1)	
>21 days	62 (8.4)	38 (5.6)	24 (42.1)	
**Inotropic or vasopressors**	311 (42.3)	286 (42.1)	25 (43.9)	0.80
**ACEi/ARB**	228 (31.0)	219 (32.3)	9 (15.8)	0.01

**ICU length of stay, (median, IQR)**	8 (6–20)	8 (6–17)	27 (17–42)	<0.001
**Hospital length of stay, (median, IQR)**	58 (36–83)	59 (36–84)	46 (31–68)	<0.01

Abbreviations: ECMO; extracorporeal membrane oxygenation, ACEi; angiotensin-converting enzyme inhibitor, ARB; angiotensin II receptor blocker, ICU; intensive care units; Values are the median (interquartile range) or n (%).
